# Role of Molecular Targeted Therapeutic Drugs in Treatment of Glioblastoma: A Review Article

**DOI:** 10.1055/s-0043-57028

**Published:** 2023-04-17

**Authors:** Himanshu Singh

**Affiliations:** 1 Department of Oral and Maxillofacial Pathology and Oral Microbiology, Index Institute of Dental Sciences, Indore, Madhya Pradesh, India

**Keywords:** glioblastoma, targeted therapy, molecular targets, therapeutic drugs

## Abstract

Glioblastoma is remarkably periodic primary brain tumor, characterizing an eminently heterogeneous pattern of neoplasms that are utmost destructive and threatening cancers.

An enhanced and upgraded knowledge of the various molecular pathways that cause malignant changes in glioblastoma has resulted in advancement of numerous biomarkers and the interpretation of various agents that pointedly target tumor cells and microenvironment. In this review, literature or information on various targeted therapy for glioblastoma is discussed. English language articles were scrutinized in plentiful directory or databases like PubMed, ScienceDirect, Web of Sciences, Google Scholar, and Scopus. The important keywords used for searching databases are “Glioblastoma,” “Targeted therapy in glioblastoma,” “Therapeutic drugs in glioblastoma,” and “Molecular targets in glioblastoma.”

## Introduction

Glioblastoma is the utmost common and destructive primary malignant brain tumor seen in adults including average overall survival (OS) of 10 to 20 months. 1 2 3 4 Glioblastoma comprises an eminently heterogeneous collection of protruding malignant tumors of the brain. 5

In a nutshell, the abovementioned research demonstrated that nearly all tumors suppress periodic molecular modifications eradicating core pathways engaged in the control of growth and deoxyribonucleic acid repair. It is acknowledged that glioblastomas are described by considerable intratumor and intertumor genomic heterogeneity. 6 7 8 9 10 Depending upon the findings of the Cancer Genome Atlas, there are four distinctive subdivisions of glioblastomas. These are the neural, proneural, mesenchymal, and classical subtypes. The neural subdivision illustrates 16% of glioblastoma. The neural subdivision is represented by the expression of various neuron markers like GABRA1, SLC1A5, and NEFL. The proneural subdivision demonstrates mutation in platelet-derived growth factor receptor A (PDGFRA). The classical subdivision demonstrated CDKN2A deletion and epidermal growth factor receptor (EGFR) amplification. The mesenchymal subdivision demonstrates mutations in phosphatase and tensin homolog (PTEN) and NF1 (neurofibromatosis type 1). 11

In this review, literature or information on various targeted therapy for glioblastoma is discussed. English language articles were scrutinized in plentiful directory or databases like PubMed, ScienceDirect, Web of Sciences, Google Scholar, and Scopus. The important keywords used for searching databases are “Glioblastoma,” “Targeted therapy in glioblastoma,” “Therapeutic drugs in glioblastoma,” and “Molecular targets in glioblastoma.”

## Various Molecular Targeted Therapeutics for Glioblastoma

### Receptor Tyrosine Kinases

They are types of transmembrane proteins. It contains a single transmembrane helix, extracellular ligand-binding domain, and intracellular catalytic domain. The receptor tyrosine kinase group consists of platelet-derived growth factors, fibroblast growth factor receptors (FGFRs), EGFRs, and hepatocyte growth factor receptors. Beneath typical physiological status, receptor tyrosine kinases are implicated in persisting cellular homeostasis by controlling cell–cell communication, cell proliferation, survival, differentiation, and migration. Therefore, dysregulation of the receptor tyrosine kinases pathway performs a crucial aspect in the initiation and progression of glioblastoma. 12 13 14

### Epidermal Growth Factor Receptor

Genomic interpretation identified that 57% of glioblastoma cells harbor EGFR genetic mutations. Overexpression and amplification of EGFRs were recognized in 60 and 40% of cases of primary glioblastoma accordingly. Overexpression and amplification result in fundamental receptor activation and intensify the survival, proliferation, and resistance to therapeutics of glioblastoma cells. 15 16 17 18 Different forms of genetic mutation were also recognized which include point mutations and rearrangement of EGFRs. 19

The utmost prevalent approach for targeting EGFRs is by way of the adoption of monoclonal antibodies. Various anti-EGFR antibodies have been established since cetuximab (the first chimeric antibody). Cetuximab and panitumumab do not demonstrate encouraging outcomes. Depatuxizumab and nimotuzumab demonstrate survival advantages when mixed with radiotherapy and chemotherapeutic temozolomide (TMZ) accordingly. 20 21 22

EGFRs are also aimed by prohibitions of the activity of tyrosine kinase. Different inhibitors have been graded in clinical research with the least possible or no advantage like gefitinib, erlotinib, and dacomitinib. Nonetheless, utilizing afatinib leads to an upsurge in progression-free survival (PFS) in those individuals that demonstrate overexpression of EGFRs. 23

### PDGFR

PDGFR is one of the targeted therapeutics in the glioblastoma–proneural subdivision. Gene amplification in PDGFR is observed in 15% of cases of glioblastoma. In different grades of gliomas, overexpression of PDGFR is observed and is linked with poor prognosis. Until now, various multikinase inhibitors like imatinib, sunitinib, and dasatinib have not demonstrated encouraging clinical advantages. 24 25

### MET

The hepatocyte growth factor receptor is encoded by the MET gene, which is expected to perform an influential function in the invasion, recurrence, migration, and drug resistance of glioma cells. 26 27 Approximately 30% of glioblastoma patients are represented by overexpression of MET. The usefulness of the rilotumumab antibody only had no action on restricting the advancement of glioblastoma. Clinical research of integrated antibody onartuzumab and antivascular drugs proved that there was no meaningful advantage for recurrent glioblastoma patients. Cabozantinib, an MET inhibitor, was moderately active in individuals with recurrent glioblastoma. 27 28 29 30 31

### PI3K/AKT/mTOR Pathway

It is the utmost prevalent alteration pathway in individuals with glioblastoma. PI3K activation in glioblastoma is chiefly because of the alteration of PTEN. 32 33

Buparlisib, a PI3K pan inhibitor, was also demonstrated to be incompetent in contrast to recurrent glioblastoma in research, either as an individual dose or linked with lomustine or carboplatin. 34 35

### Fibroblast Growth Factor Receptor

It is comprehensively expressed in glioblastoma, but its therapeutic worth may be confined to the limited count of individuals with FGFR-TACC fusion. In the current research, utilization of dovitinib was incompetent in increasing the survival of individuals whether linked with antivascular therapy or not. 36 37 38

### BRAF Mutation

BRAF takes part in Mek/Erk pathway activation and encourages the proliferation of the cell. BRAF alteration is noticed in different varieties of cancer and is demonstrated to be a trustworthy target. 39 40 41 42

### Neurotrophic Tyrosine Receptor Kinases

Three distinctive genes encode the neurotrophic tyrosine receptor kinases (NTRKs). These genes are NTRK3, NTRK2, and NTRK1. The NTRK gene genomic rearrangement results in the union of the gene, which may provoke the TRK pathway activation. This gene fusion occurrence is rarely seen in glioblastoma. Entrectinib was competent in the therapeutics of infantile glioblastoma. Larotrectinib was administered in a lady with infantile glioblastoma and the therapeutic result was noteworthy. 43 44 45 46

### The Retinoblastoma Pathway

The cell cycle regulation of the retinoblastoma protein (pRB) pathway is reciprocated because of CDK4/6 amplification, CDKN2A/B homozygous deletion, and modification of the RB1 gene. In phase II research, palbociclib has shown an unsatisfying outcome. Ribociclib was also incompetent. 47 48 49

### Proteasome

Proteasome encourage apoptosis by controlling p53, which alarmingly controls the cell cycle and alters drug resistance. Presently, various clinically recognized proteasome inhibitors include ixazomib, bortezomib, and marizomib. Bortezomib when combined with vorinostat shows inadequate results in recurrent glioblastoma. But when bortezomib is mixed with definitive radiotherapy, it shows hopeful survival rates and is well accepted. Disulfiram has advantageous blood–brain barrier penetration competence and improved drug resistance to utilize its antitumor outcome in recently diagnosed glioblastoma and recurrent glioblastoma models. 50 51 52 53 54

### Vascular Endothelial Growth Factor

Glioblastoma is described by irregularity in vascular proliferation. The vascular endothelial growth factor (VEGF) is eminently expressed in glioblastoma and advocates the anomalous proliferation of tumors. VEGFR1 and VEGFR2 pathways are recommended as a significant determinant of tumor survival in glioblastoma. 55 Bevacizumab is attached to endothelial cells and suppresses angiogenesis. In phase II research, bevacizumab demonstrates meaningful anti-glioma and biological activity, increased OS, and radiation response rate in recently diagnosed cases of glioblastoma and recurrent glioblastoma. It is also observed that bevacizumab in phase III clinical research substantially enhances PFS. 56 57 58 59 60

Bevacizumab along with TMZ demonstrates great competence and resistance. Etoposide shows an identical outcome to bevacizumab monotherapy, but etoposide displays higher toxicity. 61 62 63 Additional VEGF such as cediranib has demonstrated meaningful potency in phase II clinical research of recurrent glioblastoma. It is observed that cediranib advocates blood perfusion and extended the OS in recently diagnosed cases of glioblastoma. 64 65

Axitinib could be a promising consolidation ally with immunotherapy. Additional inhibitors such as aflibercept also downregulate the VEGF activity. 66 67

### Integrin

Integrins perform in signal transduction participating in various cellular processes. Integrins also arbitrate cellular transmission inside the extracellular matrix throughout motility, invasion, migration, angiogenesis, and adhesion. In endothelial cells, integrins αvβ5 and αvβ3 are eminently expressed and recognized as therapeutic targets in glioblastoma. 68 69

### Programmed Cell Death Protein 1

One approach to cancer immunotherapy is to forbid the communication among programmed cell death protein 1 (PD-1) on T cells and PD-1 ligand on host or tumor cells. Pembrolizumab has inadequate effectiveness in earlier therapeutics of glioblastoma, exclusive of those cases with definitive mismatch repair defects. 70 71 72 73 Nivolumab, when mixed with bevacizumab and chemoradiotherapy in recently diagnosed glioblastoma individuals along with MGMT promoter unmethylation, was unsuccessful. 74

### Lymphocyte-Activation Gene 3

Lymphocyte-activation gene 3 (LAG-3) results in an immune outbreak of tumor cells. LAG-3 is mainly seen in activated immune cells. LAG-3 is consistently expressed in T cells. Therefore, LAG-3 prohibitor evolves to a pleasant immune modulating agent only or in association with additional immune checkpoint inhibitors. In glioblastoma, LAG-3 is expressed along with CD8A, suggesting that LAG-3 targeted therapy in glioblastoma with sufficient CD8+ T cell infiltration may be hopeful. 75 76 77 78 79

### CD73

The nasal application of cationic nanoemulsion when blended with CD73-siRNA conferred hopeful anti-CD33 outcomes in glioblastoma model. 80

### V-Domain Immunoglobulin Suppressor of T Cell Activation

It has been originally acknowledged for its meaningful appearance in immunosuppression. V-domain immunoglobulin suppressor of T cell activation (VISTA) complexly and reciprocally perform as ligand and receptor in the positive and negative control of cancer immunity. 81 82 83 IgSF11 (immunoglobulin superfamily 11 gene), a VISTA ligand, demonstrates raised expression notably in high-grade glioma and corresponds with poor prognosis, implying the promising prognostic significance of IgSF11 and VISTA. 84

### CD70

CD70 is eminently overexpressed in cells of recurrent glioma in comparison to ordinary tissue and is linked with inadequate survival. Therefore, CD70 is suggested to bring about T cell apoptosis or debilitation and initiate regulatory T cells to intercede immunosuppression. 85 86

### Tumor-Associated Macrophage Therapy

Minocycline could restrain the expression of microglial matrix metalloproteinases and weaken the glioma intrusion. In addition, cyclosporine A demonstrated effectiveness in debilitating the angiogenesis and survival of glioma by restraining the microglia infiltration. Propentofylline was also demonstrated to lower the growth of tumors in glioblastoma by precisely targeting microglia. 87 88 89 90

## Conclusion

The prediction of glioblastoma stays worse and poor regardless of radiotherapy, aggressive surgery, and chemotherapies. Furthermore, numerous innovative introductions in elementary and translational researches were made in recent times. Various targeted therapies are being extensively investigated in various clinical researches. Promising advancement in glioblastoma therapeutics will apparently depend on collection of correct association of various targeted agents collectively with different multimodal therapy.
